# Survival and associated risk factors in patients with diabetes and amputations caused by infectious foot gangrene

**DOI:** 10.1186/s13047-017-0243-0

**Published:** 2018-01-04

**Authors:** Yu-Yao Huang, Cheng-Wei Lin, Hui-Mei Yang, Shih-Yuan Hung, I-Wen Chen

**Affiliations:** 1 Division of Endocrinology and Metabolism, Department of Internal Medicine, Chang Gung Memorial Hospital, Chang Gung University, 5, Fuxing St., Guishan Dist, Taoyuan City, 333 Taiwan; 2 Department of Medical Nutrition Therapy, Chang Gung Memorial Hospital, Chang Gung University, 5, Fuxing St., Guishan Dist, Taoyuan City, 333 Taiwan

**Keywords:** Diabetes, Infectious foot gangrene, Survival, Lower-extremity amputation

## Abstract

**Background:**

Infectious gangrene of the foot is a serious complication of diabetes that usually leads to a certain level of lower-extremity amputation (LEA). Nevertheless, the long-term survival and factors associated with mortality in such patients have yet to be elucidated.

**Methods:**

A total of 157 patients with type 2 diabetes who received treatment for infectious foot gangrene at a major diabetic foot center in Taiwan from 2002 to 2009 were enrolled, of whom 90 had major LEAs (above the ankle) and 67 had minor LEAs (below the ankle). Clinical data during treatment were used for the analysis of survival and LEA, and survival was tracked after treatment until December 2012.

**Results:**

Of the 157 patients, 109 died, with a median survival time of 3.12 years and 5-year survival rate of 40%. Age [hazard ratio 1.04 (95% confidence interval 1.01–1.06)], and major LEA [1.80 (1.05–3.09)] were independent factors associated with mortality. Patients with minor LEAs had a better median survival than those with major LEAs (5.5 and 1.9 years, respectively, *P* < 0.01). An abnormal ankle-brachial index was an independent risk factor [odds ratio 3.12 (95% CI 1.18–8.24)] for a poor outcome (major LEA) after adjusting for age, smoking status, hypertension, major adverse cardiac events, and renal function.

**Conclusions:**

Efforts to limit amputations below the ankle resulted in better survival of patients with infectious foot gangrene. An abnormal ankle-brachial index may guide physicians to make appropriate decisions with regards to the amputation level.

## Background

Foot gangrene, defined as dead tissue in the foot resulting from inadequate blood flow supply, is one of the manifestations of critical limb ischemia [1]. It can be caused by obstructed peripheral circulation or bacterial infections [2]. Foot ulcers in patients with diabetes are at an increased risk of foot gangrene, mainly due to peripheral arterial disease (PAD) [3] and foot infections [4]. Foot gangrene can be classified into two types: (1) dry gangrene with ischemic tissue but no infection, and (2) wet gangrene with both infectious and necrotic tissue [2]. Infectious foot gangrene (wet gangrene) is both a limb- and life-threatening disease. Proper medical treatment with antibiotics and wound dressing may not be effective without timely surgery to remove the necrotic and infectious tissue [2].

Previous reports have shown that patients with diabetes-related amputations have a high risk of mortality, with a 5-year survival rate of 40–48% regardless of the etiology of the amputation [5–7]. The risk factors associated with survival have been investigated in patients with diabetic foot ulcers [8, 9] but rarely in diabetic amputees [7]. Survival analysis is also lacking in patients with diabetes and infectious foot gangrene who need more medical attention for both critical limb ischemia and limb-threatening infections [10]. Therefore, the aim of this study was to investigate the survival outcomes of patients with diabetes and amputations due to infectious foot gangrene and the factors associated with the prognosis.

## Methods

### Patients and hospital care

A total of 157 patients with type 2 diabetes and infectious foot gangrene who were admitted to the diabetic foot care center of Chang Gung Memorial Hospital in Taiwan from 2002 to 2009 were enrolled. This study was approved by the Institutional Review Board of Chang Gung Memorial Hospital (no. 104-1401B). All of the patients received multidisciplinary care including glycemic control, antibiotic treatment and wound dressing. Surgical interventions to remove necrotic tissue or limbs were determined according to the consensus of a team of physicians taking into account the risk of mortality due to co-morbidities and clinical status or an obviously poor likelihood of preserving the foot (due to large tissue defects, uncontrolled infections or severe artery occlusion with no possibility of revascularization) [11, 12].

All of the enrolled patients received some degree of gangrene tissue removal, including 90 with major lower-extremity amputation (LEAs) (above the ankle) and 67 with minor LEAs (including digital amputation or tarsal-metatarsal amputation but not the ankle area).

### Data collection

Survival time was calculated from the discharge date to December 31, 2012, or death. Information of death was obtained from the National Health Insurance database.

Clinical data were collected during the admission for foot gangrene treatment. Age, duration of diabetes and HbA1c were recorded as continuous variables. Gender, smoking status, underlying co-morbidities (hypertension, major adverse cardiac events (MACEs), and renal status), ankle-brachial index (ABI) and amputation level were recorded as categorical variables. Smokers were defined as currently smoking with at least one cigarette per day, and renal status was defined according to the National Kidney Disease Outcomes Quality Initiative (NKDOQI) guidelines as follows: normal renal function with an estimated glomerular filtration rate (eGFR) calculated according to the modification of diet in renal disease equation of ≥60 ml/min; moderate chronic kidney disease (CKD) with an eGFR <60 ml/min but not requiring dialysis treatment; and dialysis status as the continuous need for renal replacement therapy. ABI was calculated by measuring the ratio of the blood pressure of the lower leg to the blood pressure in the arm according to the 2011 American Heart Association guidelines for peripheral arterial disease [13], and an abnormal ABI was defined as either ≤0.9 or >1.4.

### Statistics

Kaplan-Meier method was used to estimate the survival probability for differing survival times (time to event). Each variable was entered into a multivariate Cox regression model to identify the independent risk factors associated with mortality. Differences in survival and clinical data were calculated between the minor and major LEA groups using the Kaplan-Meier method and log rank test for survival, the Student’s *t* test for continuous variables, and Pearson’s chi-square test for categorical variables. In addition, multivariate logistic regression analysis was performed including age, smoking status, hypertension, MACEs, renal function and PAD to identify the independent risk factors predicting the outcomes of the patients with major LEAs.

All statistical analyses were performed using the Statistical Package for the Social Sciences (SPSS for Windows, version 19.0, IBM Corp., Armonk, NY). Statistical significance was defined as a *P* value <0.05.

## Results

### Demographic data

The mean age of the enrolled patients was 66.8 years, and 63.1% of the patients were male (Table 1). Overall, 70.7% of the patients had underlying hypertension, 33.1% had MACEs, and 25.5% were undergoing chronic dialysis for end-stage renal disease (Table 1). PAD was noted in 60% of the patients, with 11 patients having an ABI > 1.4 and 93 an ABI ≤ 0.9.

**Table 1:** Demographics and clinical characteristics of the patients with diabetes and infectious foot gangrene and risk factors for mortality in a Cox proportional hazards model (Total number: 157)

Characteristic	Mean (standard deviation) or Number (%)		Multivariate^+^ analysis	
			Mortality HR (95% CI)	*P* value
Age (years)	66.8	(±10.8)	1.04^*^(1.01–1.06)	0.01
Gender				0.37
Female	58	(36.9%)	1	
Male	99	(63.1%)	0.79 (0.46–1.33)	
Diabetes duration (years)	12.8	(±8.4)	1.14 (0.92–1.43)	0.24
Smoking status	34	(21.7%)	1.48 (0.73–3.01)	0.28
Hypertension	111	(70.7%)	1.06 (0.63–1.80)	0.83
Major adverse cardiac events	52	(33.1%)	1.48 (0.88–2.50)	0.14
Abnormal ABI	104	(66.2%)	1.28 (0.67–2.42)	0.46
Renal function				
Normal renal function (eGFR ≥60)	58	(36.9%)	1	
Chronic kidney disease (eGFR <60)	59	(37.6%)	0.87 (0.48–1.59)	0.66
Dialysis	40	(25.5%)	2.09^^^(0.99–4.41)	0.05
HbA1c (%)	9.4	(±2.7)	1.02 (0.92–1.13)	0.71
Limb amputation status				
Minor LEA	67	(42.7%)	1	
Major LEA	90	(57.3%)	1.80^*^(1.05–3.09)	0.03

^*^Significance: *P* value <0.05

^^^Borderline significance: *P* value = 0.05

^+^Including continuous variables of age, diabetes duration, and HbA1c; and categorical variables of gender, smoking status, hypertension, major adverse cardiac events, ankle-brachial index, renal function and LEA status

*HR* hazard ratio, *ABI* ankle-brachial index, *eGFR* estimated glomerular filtration rate, *LEA* lower-extremity amputation

### Survival analysis

Of the 157 enrolled patients, 109 died during a mean of 3.7 years observation period (range: 0–10.5 years). The 5-year survival rate was 40% with a median survival time of 3.12 years and a crude mortality rate of 187.64 per 1000 patient-years.

**Fig. 1:**
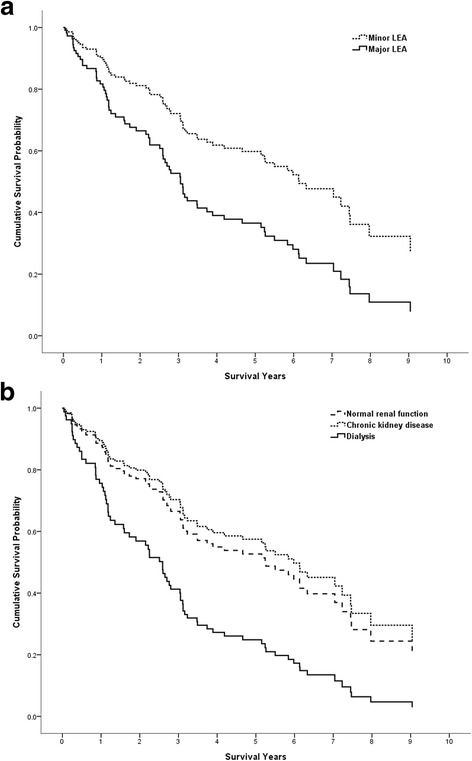
Adjusted survival curve from a Cox proportional hazard model according to limb amputation status and renal function status. **a** Compared to the minor LEA group, the adjusted hazard ratio for mortality was 1.80 (95% CI 1.05–3.09, *P* = 0.03) for those with major LEAs. **b** Compared to the normal renal function group, the adjusted hazard ratio for mortality was 0.87 (95% CI 0.48–1.59, *P* = 0.66) for those with CKD and 2.09 (95% CI 0.99–4.41, *P* = 0.05) for those undergoing dialysis. Covariates included continuous variables of age, diabetes duration, and HbA1c; and categorical variables of hypertension, major adverse cardiac events, renal function, ABI value and limb amputation status

In multivariate Cox regression analysis with the variables listed in Table 1, age [adjusted hazard ratio (aHR) 1.04 (95% confidence interval (CI) 1.01–1.06)] and major LEA [aHR 1.80 (95% CI 1.05–3.09)] were independent risk factors associated with long-term mortality (Table 1). Although there was no statistical difference in renal function, the patients undergoing dialysis had a 2-fold higher risk of mortality compared to those with normal renal function [aHR 2.09 (95% CI 0.99–4.41)]. In contrast, the survival curves of the patients with or without CKD were similar (Fig. 1 and Table 1). Figure 1 shows an early separation of survival curves between the patients with minor and major LEAs. Compared to the patients with minor LEAs, those with major LEAs had an 80% higher risk of mortality. The median survival time of the patients with minor LEAs was 5.5 years, compared to 1.9 years for those with major LEAs (*P* < 0.01) (Table 2).

**Table 2:** Clinical characteristics of the patients with diabetes and infectious foot gangrene with different LEA status

Characteristic	Mean (standard deviation) or Number (%)				
	Minor LEA (*n* = 67)		Major LEA (*n* = 90)		*P* value
Death	38		48		
Median survival time (years)	5.5 (95% CI: 2.58–8.42)		1.9 (95% CI: 1.15–2.65)		<0.01^*^
5-year survival rate	54%		30%		
Age (years)	65.8	(±11.7)	67.6	(±10.1)	0.32
Gender					0.56
Female	23	(34.3%)	35	(38.9%)	
Male	44	(65.7%)	55	(61.1%)	
Diabetes duration (years)	11.9	(±8.9)	13.5	(±8.1)	0.24
Smoking status	18	(26.9%)	16	(17.8%)	0.17
Hypertension	44	(65.7%)	67	(74.4%)	0.23
Major adverse cardiac events	17	(25.4%)	35	(38.9%)	0.08
Abnormal ABI	32	(47.8%)	72	(80.0%)	<0.01^*^
Renal function					0.09
Normal renal function (eGFR ≥60)	31	(46.3%)	27	(30.0%)	
Chronic kidney disease (eGFR <60)	23	(34.3%)	36	(40.0%)	
Dialysis	13	(19.4%)	27	(30.0%)	
HbA1c (%)	9.7	(±2.5)	9.0	(±2.9)	0.14
Hospitalization duration (days)	48.2	(±34.7)	36.9	(±25.2)	0.03^*^

^*^Significance: *P* value <0.05

^+^Including continuous variables of age and diabetes duration; and categorical variables of gender, smoking status, hypertension, major adverse cardiac events, ankle-brachial index, and renal function

*LEA* lower-extremity amputation, *ABI* ankle-brachial index, *eGFR* estimated glomerular filtration rate

### Comparisons between the minor and major LEA groups

The patients with major LEAs had obviously abnormal ABI values at treatment (80% vs. 47.8%, *P* < 0.01), and also relatively higher rates of MACEs and renal dysfunction although without reaching statistical significance. A longer hospital stay has been observed in patients with minor LEAs than those with major LEAs (48.2 vs. 36.9 days, respectively, *P* = 0.03).

Since LEA status had an impact on survival, we further analyzed the factors predicting the level of LEA (Table 3). After adjusting for clinical data including age, smoking status, hypertension, MACEs, and renal function in multivariate logistic regression analysis, an abnormal ABI value at treatment was an independent factor predicting a major LEA [odds ratio 3.12 (95% CI 1.18–8.24), *P* = 0.02].

**Table 3:** Multivariate logistic regression analysis of risk factors to predict major LEA in the patients with infectious foot gangrene

Factors^+^	Interpretation	Odds ratio	95% confidence interval	*P* value
Abnormal ABI	Yes vs. No	3.12^*^	1.18–8.24	0.02
Age	Every 1 year increment	0.97	0.93–1.02	0.21
Diabetes duration	Every 1 year increment	0.99	0.94–1.04	0.66
Smoking status	Yes vs. No	0.54	0.19–1.53	0.25
Hypertension	Yes vs. No	1.28	0.53–3.10	0.59
MACE history	Yes vs. No	1.13	0.45–2.80	0.80
CKD (eGFR < 60)	vs. normal renal function (eGFR ≥60)	1.81	0.74–4.46	0.20
Dialysis	vs. normal renal function (eGFR ≥60)	2.51	0.71–8.85	0.15
HbA1c	Every 1% increment	0.95	0.81–1.13	0.58

^*^Significance: *P* value <0.05

^+^Including the continuous variable of age, diabetes duration and HbA1c; and categorical variables of smoking status, hypertension, major adverse cardiac events, ankle-brachial index, and renal function

*LEA* lower-extremity amputation, *ABI* ankle-brachial index, *MACE* major adverse cardiac event, *CKD* chronic kidney disease, *eGFR* estimated glomerular filtration rate

## Discussion

In this study, the patients with diabetes and infectious foot gangrene related amputees had a 5-year survival rate of 40% after treatment. This survival rate is not better than other studies on diabetic amputees with various etiologies, ranging from a 5-year survival rate of around 40% reported by Tseng et al. [5], 44% by Hambleton et al. [6], 47.7% by Lavery et al. [7] and 64%–84.2% by Izumi et al. [14]. The mean age of our patients was 66.8 years, which is similar to that in the studies by Lavery et al. and Tseng et al. (64.8 and 66.6 years, respectively) but older than that in Izumi et al.’s study (53.8 years). A more recent study reported a 30% 5-year survival rate in patients with diabetes and major LEA, which is similar to our subgroup patients with major LEA [15].

In addition to the age factor, another independent factor associated survival is the outcome of major LEA. Major LEAs resulted in a worse survival prospect than minor LEAs, which is consistent with other survival studies on diabetic amputees [6, 7, 14]. This may be due to major LEA-related disabilities such as limited mobility [16], bed-ridden status [17], and higher risk of falls [18]. Furthermore, a major LEA by itself may increase the risk of cardiovascular disease because of behavioral changes, psychosocial stress, and increases in inflammation and insulin resistance [19].

Prompt major LEA for infectious foot gangrene is sometimes suggested by physicians to control foot infection and also to allow for early rehabilitation such as wearing prostheses and ambulation to avoid restricted activity-related disabilities during hospitalization [20]. However, the rate of successful rehabilitation after major LEA is low because of poor motivation and multiple co-morbidities, and only around 37% of patients who undergo a major LEA recover to pre-amputation mobility [21].

The results of this study suggest that even though factors such as age and dialysis status beyond our control, avoiding major LEAs could prolong the survival of patients with diabetes and infectious foot gangrene. However, limb preservation is challenging for those patients because of difficulty in wound healing, severe comorbidities, and the high risk of progressive sepsis [22]. Furthermore, the aggressive limb preservation treatments may include revascularization procedures, advanced wound dressing, off-loading, and meticulous medical cares [23]. That also explains longer hospital stays in patients with minor LEA. It is also troubling to directly perform a major LEA because of the resistance of patients and their family [24].

In this and our previous study, we showed that the ABI value could be used as a predictor for the outcome of minor or major LEAs [25]. However, it could not predict survival prospect in this population. To date, no study has investigated the influence of the ABI on survival rate in diabetic amputees. However, ABI value has been reported to be an independent risk factor for survival in patients with diabetes and foot ulcers regardless of the LEA status [8, 9]. The ABI has also been reported to be a predictor of MACEs because PAD is thought to be a manifestation of systemic atherosclerosis rather than only localized disease [26, 27]. This suggests patients with diabetes and infectious foot gangrene are at risk of systemic atherosclerosis in addition to limb PAD. Accordingly, for the patients with foot gangrene and a normal ABI, it is more beneficial to preserve more distal limbs by aggressive treatment, because if the amputation level is limited to below the ankle, the long-term survival is better.

Another interesting factor associated survival with borderline significance was the renal function status. A reduced eGFR has been reported to be associated with increased risks of death, cardiovascular events, and hospitalization [28, 29]. Lavery et al. analyzed patients with diabetes who underwent LEA and found that those undergoing dialysis had the worst survival rate, followed by those with CKD and normal kidneys [7]. In the current study, however, the impact of CKD on long-term survival was not as obvious, suggesting that the impact on health of foot gangrene and subsequent LEA outweighed the impact of CKD itself. However, in the patients requiring dialysis, end-stage renal disease still influenced the long-term survival.

This study is limited by the single center and retrospective design. In addition, although we evaluated lower limb circulation, the rate of revascularization was relatively low because of clinical concerns over the risk of kidney injury, intravascular procedure complications in the patients with sepsis, and difficulty of the procedure. Therefore, the revascularization data in this study had little power in survival analysis. Further studies are needed to elucidate the importance of revascularization in patients with infectious foot gangrene.

## Conclusions

The patients with diabetes and amputations caused by infectious foot gangrene in this study had a limited 5-years survival rate of around 40%. Age and LEA level were risk factors associated with mortality. The ABI may assist with appropriate decision making with regards to limb amputation level.

## Abbreviations

ABI: Ankle brachial index
aHR: Adjusted hazard ratio
CKD: Chronic kidney disease
eGFR: Estimated glomerular filtration rate
LEA: Lower-extremity amputation
MACE: Major adverse cardiac event
PAD: Peripheral arterial disease
